# Descriptive analysis of a comparison between lung ultrasound and chest radiography in patients suspected of COVID-19

**DOI:** 10.1186/s13089-021-00215-9

**Published:** 2021-02-26

**Authors:** Giovanni Volpicelli, Luciano Cardinale, Thomas Fraccalini, Marco Calandri, Clara Piatti, Carlotta Geninatti, Giuseppe Stranieri

**Affiliations:** 1 Department of Emergency Medicine, San Luigi Gonzaga University Hospital, Torino, Italy; 2 Department of Oncology, Radiology Unit, San Luigi Gonzaga University Hospital, Torino, Italy

## Abstract

**Background:**

Lung ultrasound (LUS) and chest radiography (CXR) are the most used chest imaging tools in the early diagnosis of COVID-19 associated pneumonia. However, the relationship between LUS and CXR is not clearly defined. The aim of our study was to describe the comparison between LUS interpretation and CXR readings in the first approach to patients suspected of COVID-19.

**Methods:**

In the time of the first COVID-19 pandemic surge, we prospectively evaluated adult patients presenting to an emergency department complaining of symptoms raising suspicion of COVID-19. Patients were studied by LUS and only those performing also CXR were analyzed. All the patients performed viral reverse transcriptase-polymerase chain reaction (RT-PCR). LUS studies were classified in 4 categories of probabilities, based on the presence of typical or alternative signs of COVID-19-associated interstitial pneumonia. Accordingly, the CXR readings were retrospectively adapted by 2 experts in 4 categories following the standard language that describes the computed tomography (CT) findings. Patients were divided in two groups, based on the agreement of the LUS and CXR categories. Results were also compared to RT-PCR and, when available, to CT studies.

**Results:**

We analyzed 139 cases (55 women, mean age 59.1 ± 15.5 years old). The LUS vs CXR results disagreed in 60 (43.2%) cases. RT-PCR was positive in 88 (63.3%) cases. In 45 cases, a CT scan was also performed and only 4 disagreed with LUS interpretation versus 24 in the comparison between CT and CXR. In 18 cases, LUS detected signs of COVID-19 pneumonia (high and intermediate probabilities) while CXR reading was negative; in 14 of these cases, a CT scan or a RT-PCR-positive result confirmed the LUS interpretation. In 6 cases, LUS detected signs of alternative diagnoses to COVID-19 pneumonia while CXR was negative; in 4 of these cases, CT scan confirmed atypical findings.

**Conclusion:**

Our study demonstrated a strong disagreement between LUS interpretation and CXR reading in the early approach to patients suspected of COVID-19. Comparison with CT studies and RT-PCR results seems to confirm the superiority of LUS over a second retrospective reading of CXR.

## Introduction

The COVID-19 pandemic poses unprecedented challenges to the health care systems even in the most advanced and rich countries. During the pandemic surge, there is the need to isolate new cases and diagnose promptly interstitial pneumonia, the most common complication of the SARS-CoV-2 infection [1]. Chest imaging plays a crucial role in the frontline. Among the chest imaging tools of common use, chest computed tomography scan (CT) represents the gold standard for diagnosing pneumonia. In recent studies, CT showed to be highly sensitive in revealing the early signs of interstitial pneumonia even on asymptomatic COVID-19 patients and on patients with initial negative reverse transcriptase-polymerase chain reaction (RT-PCR) swab test [2, 3]. However, a systematic application of CT during the pandemic surge is not feasible for many reasons, including the biological and economic cost, the unavailability in scarce resource areas and the increase in the risk of in-hospital cross-infections when infected patients are moved to the radiology facilities. The recommendation to limit the use of CT studies in the time of a pandemic surge is also supported by societal guidelines [4, 5]. Lung ultrasound (LUS) and chest radiography (CXR) are valid alternatives to be applied in the frontline [1, 6–14]. Particularly, LUS demonstrated highly feasible and sensitive for COVID-19 pneumonia, with a specificity that increases during the peaks of prevalence [15]. The correct application of LUS depends also on the consideration of different degrees of likelihood for COVID-19 pneumonia and the combination of LUS patterns with the patient’s clinical phenotype at presentation to the emergency department (ED) [1, 7, 14]. Instead, the application of CXR in the first diagnosis of COVID-19 pneumonia is limited by a low sensitivity, even if this latter seems to improve in cases with a longer duration of symptoms [10, 16].

To the best of our knowledge, there are no studies comparing LUS and CXR in the first diagnosis of COVID-19 pneumonia, when a systematic classification in patterns of probability is applied. The main aim of our study was to describe the agreement between LUS interpretation and CXR reading in symptomatic patients suspected of COVID-19 who presented to the ED during the peak of COVID-19 pandemic in the spring 2020. A second aim was to describe the combination with the results of CT scan and RT-PCR, in cases showing LUS/CXR disagreement.

## Methods

This was a monocenter observational descriptive study performed at San Luigi Gonzaga University Hospital in Torino, Italy. We received the ethical approval from the local ethical committee. During the first surge of the COVID-19 pandemic, from March 10 to May 15, adult patients (≥ 18 years old) presenting to the ED complaining of symptoms raising suspicion of SARS-CoV-2 infection and studied by LUS at presentation, were enrolled. Clinical suspicion was based on the following symptoms: at least one major criteria between fever > 37.5° C, cough, dyspnea, anosmia and/or ageusia; or at least two minor criteria between sore throat, bilateral conjunctivitis, deep weakness, rhinorrhea, headache, diffuse muscle-skeletal pain, gastrointestinal symptoms (diarrhea, nausea, vomit); (2) age ≥ 18-year-old. Symptoms were to last for at least 3 days in the absence of an alternative more probable diagnosis. Exclusion criteria were: (1) a previous diagnosis of COVID-19 pneumonia; (2) a previous pneumonectomy, pleurodesis or history of fibrothorax.

All patients were prospectively studied by bedside LUS at presentation for signs of COVID-19 pneumonia. Decision about performing a CXR study was taken by the physician in charge, blinded to the LUS evaluation and for clinical reasons independent from the study protocol. Only patients who performed both LUS and CXR were analyzed. Performance of a CT scan was decided by the physician in charge for clinical reasons independent from the study protocol, after having considered both the results of LUS and CXR. All the enrolled patients were submitted to RT-PCR swab test immediately after presentation.

### Clinical characteristics

The clinical characteristics at presentation were simplified by considering respiratory symptoms and the clinical history. We identified 3 different clinical phenotypes based on pre-existing chronic cardiac or respiratory diseases that may entail the possibility of confounding ultrasound and radiologic signs (*mixed phenotype*), and on the presence (*severe phenotype*) or absence (*mild phenotype*) of signs and/or symptoms of respiratory failure. The list of significant chronic conditions included: severe chronic obstructive pulmonary disease, pulmonary fibrosis, lung cancer, heart failure, or cor pulmonale. Respiratory failure was determined according to the presence of dyspnea, either objective or self-reported to the attending physician at admission, and/or desaturation defined as either a PaO_2_/FiO_2_ < 300 mmHg or Sat.O_2_/FiO_2_ < 357.

### Lung ultrasound

For many years, LUS has become a routine diagnostic tool in our institution. The exam was performed by experienced operators with documented previous experience in bedside LUS in the diagnosis of common lung pathologies seen in emergency and critical care. The operator assigned the LUS pattern at patient’s presentation being aware of symptoms but blinded to any other exam and diagnostic tool. Further details about the technique are reported in previous publications [1, 7]. An Esaote MyLab and a Mindray TE7 equipped with convex transducers (3.5–6.0 MHz) were used. The focus was placed at the height of the pleural line. The depth of the image was set at 8–10 cm (according to patient’s size). The gain was regulated to maintain the homogeneity of the echoic image on the whole screen, including the bottom edge of the lung image. Patients were examined in the supine and lateral decubitus. We performed 5 scans per side by recording as many clips of a duration of 10 secs, in the following areas and the following probe orientation: anterior chest in longitudinal, lateral chest in longitudinal, lateral base in oblique, posterior paravertebral in longitudinal, posterior sub-scapular in oblique. We considered the presence of some basic LUS signs: A-lines; multiple B-lines, either separated, coalescent or “light beam” [6, 17]; regular or irregular pleural line; peripheral consolidations; large consolidation with air bronchogram; pleural effusion. The combination and distribution of the LUS signs were analyzed to assign to each exam one of 4 LUS patterns that express different levels of likelihood for COVID-19 pneumonia in the time of a high pandemic prevalence of the disease:

- *High probability* LUS pattern (HighLUS), typical of COVID-19 pneumonia showing bilateral and multifocal clusters of separated and coalescent B-lines, large echoic bands (light beams), multifocal small peripheral consolidations, alternating regular and irregular pleural line, with or without large consolidations; these clusters should appear in patchy distribution, alternating abruptly with normal A-lines patterns ("spared areas”);
- *Intermediate probability* LUS pattern (IntLUS), less typical pattern of COVID-19 pneumonia, which includes unilateral isolated clusters of B-lines and light beam or focal multiple B-lines, with or without small peripheral consolidations;
- *Alternative probability* LUS pattern (AltLUS), a condition more consistent with other diagnosis than COVID-19 pneumonia, such as an isolated large consolidation with dynamic air bronchograms (suggesting bacterial pneumonia) or without bronchograms (suggesting obstructive atelectasis), a large pleural effusion (either hydrostatic or inflammatory), diffuse homogeneously distributed B-lines (suggesting cardiogenic edema or diffuse fibrosis);
- *Low probability* LUS pattern (LowLUS), a normal or near-normal LUS pattern characterized by bilateral A-lines with respiratory sliding without significant B-lines.

### Chest radiography

All radiological studies were acquired as a digital radiograph in the bedside antero-posterior view using portable x-ray units (GE OPTIMA XR 200amx, General Electric Healthcare, Milwaukee WI, US) in the isolation wards following local protocols (95 kV, 1,6 mAs, no grid). The CXR images were retrospectively reviewed independently by two radiologists with long-standing experience on thoracic imaging. In case of disagreement in the reading, a consensus was reached after discussion. The findings considered were presence of few basic signs and their distribution in the two lungs. We used a standard definition of CXR alterations, according to the language approved by the Fleischner Society [18]: *ground glass opacities* (GGO), extensive blurred lung opacities, with indistinct margins of blood vessels; *reticular alterations*, visualized as a net image, formed by summation of several small linear opacities; *consolidations*, homogeneous increase in parenchymal attenuation that obscures the visualization of walls of vessels and airways. The distribution of CXR signs of interstitial pneumonia and the presence of additional signs atypical of COVID-19 pneumonia, were used to define 4 classes similar to those used to standardize CT readings [19]:

- *Typical* appearance in the presence of bilateral distribution of GGO and reticular alterations, with or without presence of limited consolidations;
- *Indeterminate* appearance when opacities were limited and monolateral or smooth and not well defined;
- *Atypical* appearance when signs alternative to COVID-19 pneumonia were predominant or isolated, like a large effusion, a large lobar consolidation with evidence of well-defined air bronchograms, clear signs of atelectasis;
- *Negative* when the CXR did not demonstrate any significant alteration.

### Computed tomography

Patients with clinical indication to CT scan were moved to the radiology unit to perform the study. A multidetector CT (GE OPTIMA 660 General Electric Healthcare, Milwaukee WI, USA) was used. The patients were in supine and head-first position and received scanning with breath held. Parameters used were: 10 0 kV; 100, mAs real-time adaptive control; layer thickness 1–2.5 mm; pitch, 1–1.5; matrix, 512 × 512. No contrast was administered. All images were transmitted to the post-processing workstation and reconstructed using high-resolution and conventional algorithms. Each study was read and interpreted by two experts with long-standing experience in chest imaging. Interpretation of CT scans was performed by radiologists not involved in CXR readings, blind to the results of LUS and CXR. Signs and nomenclature of CT scan were those recommended and reported in the Radiological Society of North America Expert Consensus document [19]. Each CT scan was assigned to one of the 4 imaging classification recommended in the consensus document:

- *Typical* appearance, in the presence of peripheral, bilateral, multifocal GGO with or without consolidation or visible lines (“crazy-paving”);
- *Indeterminate* appearance, in the presence of multifocal perihilar or unilateral GGO with or without consolidation, or very small GGO non-rounded or non-peripheral;
- *Atypical* appearance, in the presence of isolated lobar or segmental consolidation without GGO, or discrete small nodules, or lung cavitation, or smooth interlobular septal thickening with large pleural effusion;
- *Negative*, in case of no CT features suggesting pneumonia.

### Statistical analysis

Patients were grouped according to agreement or disagreement between LUS interpretation and CXR reading, following the reciprocal classification in the four categories. Continuous variables are expressed as mean with SD. Categorical variables were expressed as numbers and percentages. The normality of distribution for continuous numeric data was assessed with Shapiro–Wilk test. Non-continuous non-normally distributed variables were analyzed using Chi-Squared test or Fisher’s test in case of small numbers. Mann–Whitney test was used to compare continuous non-normally distributed variables. Unpaired T-Test in case of continuous normally distributed variables. P values below 0.05 were considered statistically significant. Statistical analysis was performed using MedCalc statistical software.

## Results

**Fig. 1:**
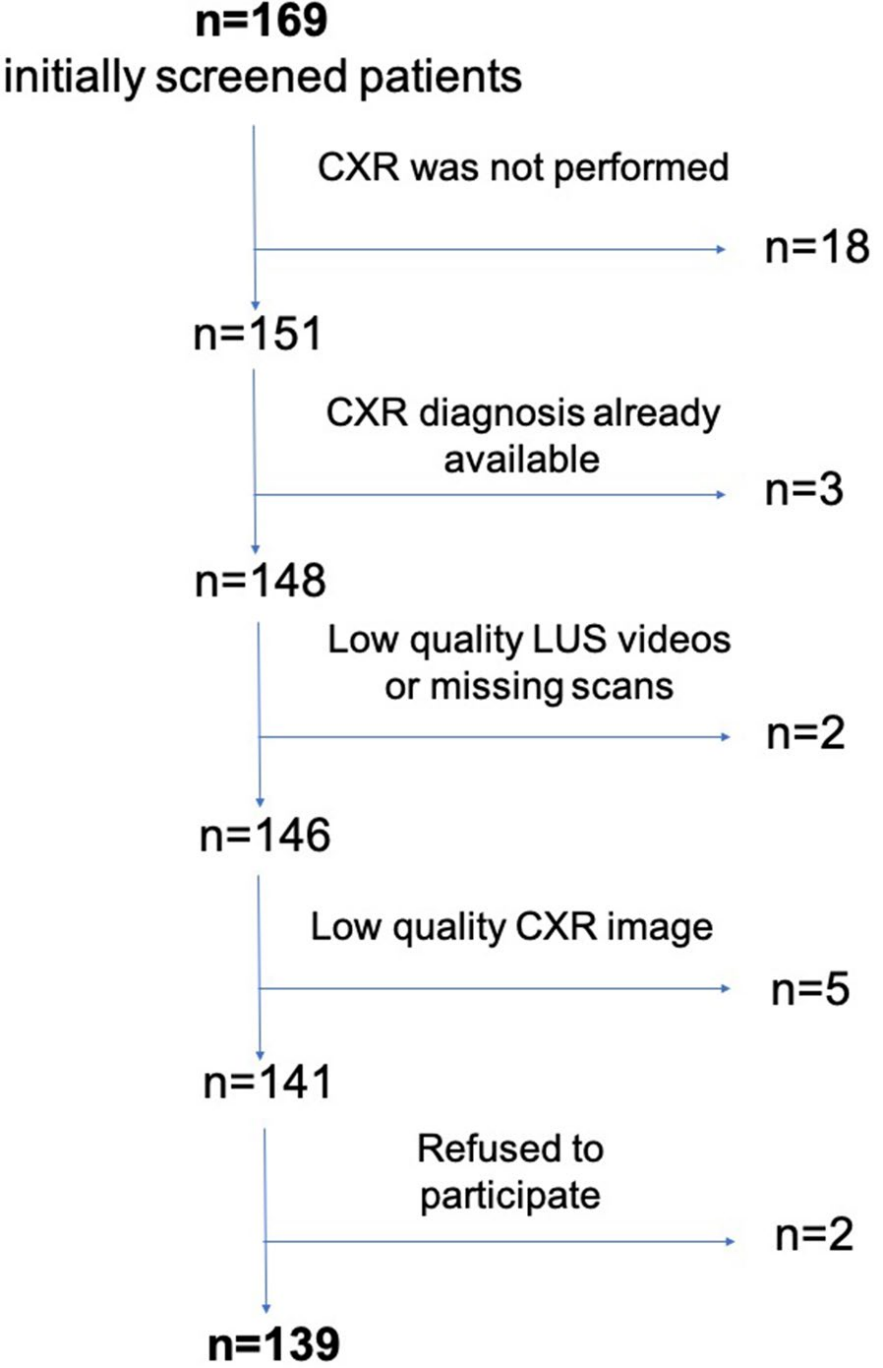
The flow chart of patients enrolled in the study

**Fig. 2:**
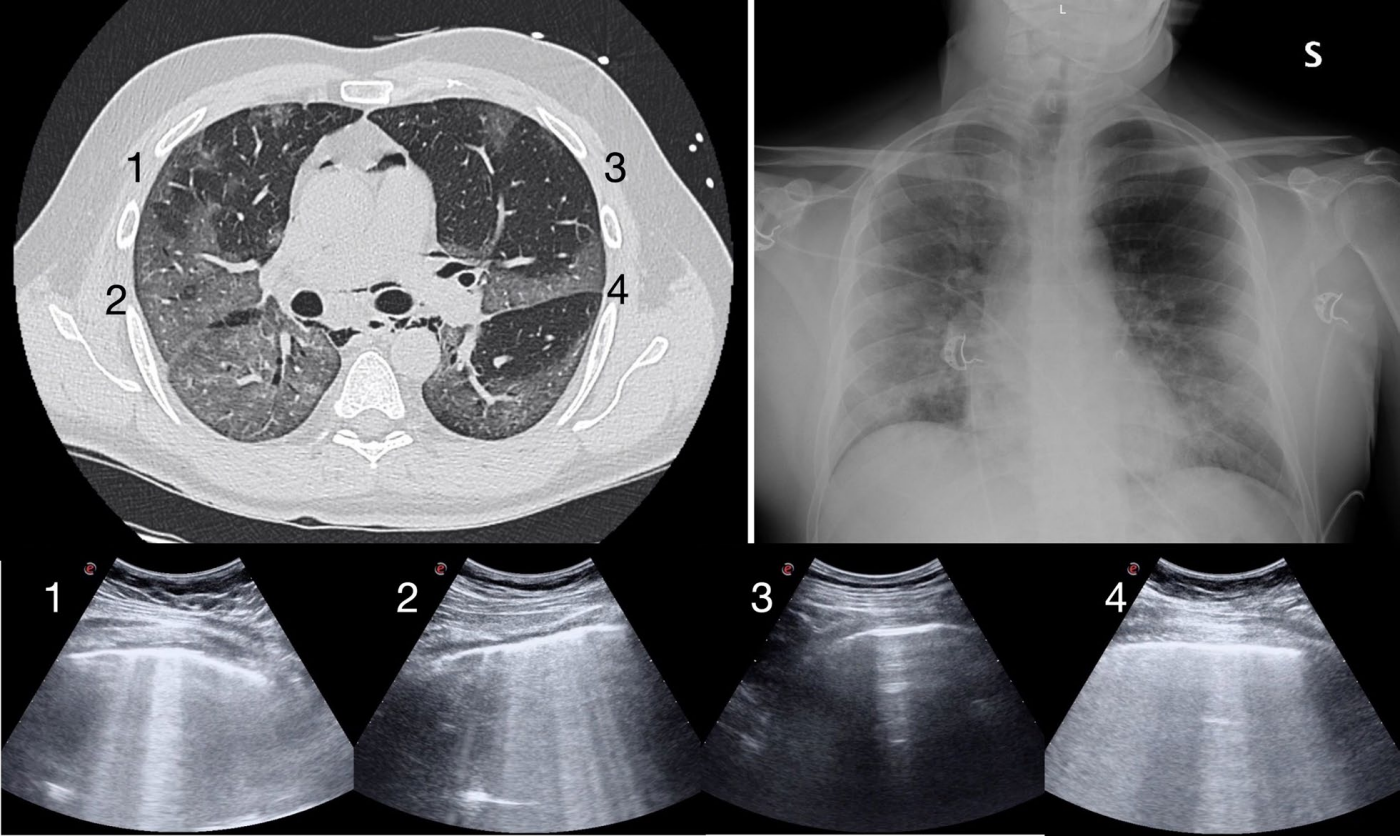
The case of an otherwise healthy 46-year-old man complaining of fever, cough and dyspnea for 7 days (*severe phenotype*). LUS and CXR were in agreement. LUS showed a *high probability pattern* for COVID-19 pneumonia. The LUS scans reported in the bottom panel show a patchy and bilateral distribution of clusters of B-lines and light beam (1, 2, 4), alternating with normal A-lines pattern (3). CXR in the upper right panel was read *typical* for the presence of bilateral GGO and reticular alterations. The corresponding CT scan in the upper left panel shows multiple GGO alterations that were considered *typical appearance*. The points numbered in the CT image correspond to the 4 LUS scans. RT-PCR was positive. Unfortunately, the patient died of COVID-19 after 20 days from admission. *LUS*   Lung Ultrasound, *CXR*  Chest Radiography, *COVID-19*  Corona Virus Disease 2019, *GGO*   Ground Glass Opacity, *CT*   Computed Tomography, *RT-PCR*  Reverse Transcriptase-Polymerase Chain Reaction

Out of 169 patients enrolled in the study, 139 (55 women and 84 men) with a mean age 59.1 ± 15.5 years old performed both CXR and LUS and were examined. The patient’s flow chart is represented in Fig. 1. RT-PCR confirmed the SARS-CoV-2 infection in 88 cases (63.3%). Mean number of days from onset of symptoms in the overall population was 7.05 ± 5.6 days. In 45 patients, a thoracic CT study was performed. All the CXR and CT studies were performed within 2 h from the LUS exam. After having assigned each CXR reading and LUS pattern interpretation to the four appearance and probability classes, patients were divided in two groups: 79 (56.8%) patients with agreement (Fig. 2) and 60 (43.2%) with disagreement of CXR vs LUS. Table 1 reports the patient’s characteristics in the two subgroups. No statistically significant difference was found between the two groups, apart from the age that resulted higher in the group with LUS vs CXR disagreement (62.8 ± 14.2 vs 56.4 ± 17.6; *p* = 0.022).

**Table 1:** Patients’ characteristics according to agreement between CXR readings and LUS patterns

	Concordant cases (*n* = 79)	Discordant cases (*n* = 60)	P
Age (mean ± SD, years)	56.4 ± 17.6	62.8 ± 14.2	0.022
Female sex (*n*)	37 (46.8%)	18 (30.0%)	0.055
Days symptoms onset (mean ± SD)	7.53 ± 5.7	6.43 ± 5.5	0.256
Mild phenotype (*n*)	35 (44.3%)	32 (53.3%)	0.309
Severe phenotype (*n*)	35 (44.3%)	19 (31.7%)	0.161
Mixed phenotype (*n*)	9 (11.4%)	9 (15.0%)	0.613

When the two LUS classifications HighLUS and IntLUS are mixed together and matched with both CXR *typical* and *indeterminate*, the number of cases showing disagreement was reduced to 46 (33.1%). In the 79 patients showing agreement between LUS pattern and CXR reading, the LUS diagnoses were: 28 LowLUS, 39 HighLUS, 5 IntLUS and 7 AltLUS. Table 2 reports the assignments of LUS patterns together with CXR and CT readings and results of RT-PCR in the subgroup with disagreement.

**Table 2:** Distribution of LUS patterns, CXR readings (*n*), CT findings (*n*) and RT-PCR results (*n*) in 60 cases with disagreement between LUS and CXR, out of 139 patients suspected of COVID-19

LUS pattern	CXR positive	CXR negative	CXR indeterm	CXR atypical	CT typical	CT negative	CT indeterm	CT atypical	RT-PCR positive	RT-PCR negative
HighLUS (*n* = 27)	–	8	12	7	11	0	0	0	24	3
IntLUS (*n* = 14)	2	10	–	2	0	0	3	1	8	6
AltLUS (*n* = 13)	5	6	2	–	0	1	1	6	2	11
LowLUS (*n* = 6)	0	–	3	3	0	2	0	0	3	3

*LUS*  Lung Ultrasound, *CXR  *Chest X-Ray, *CT* Computed Tomography of the chest, *RT-PCR*  Reverse Transcriptase-Polymerase Chain reaction, *COVID-19* Corona Virus Disease 2019, *HighLUS*  High Probability Lung Ultrasound, *LowLUS*  Low Probability Lung Ultrasound, *IntLUS* Intermediate Probability Lung Ultrasound, *AltLUS*  Alternative Probability Lung Ultrasound, I*ndeterm*  indeterminate

*CT scans*: out of the 45 CT studies, 24 disagreed with CXR reading and 4 with LUS interpretation. The 4 cases showing CT vs LUS disagreement, were: one case of *negative* CT that was interpreted AltLUS and *typical* CXR; one case of *typical* CT that was interpreted LowLUS and *negative* CXR; one case of *indeterminate* CT that was interpreted AltLUS and *typical* CXR; one case of *atypical* CT that was interpreted IntLUS but confirmed *atypical* at CXR.

**Fig. 3:**
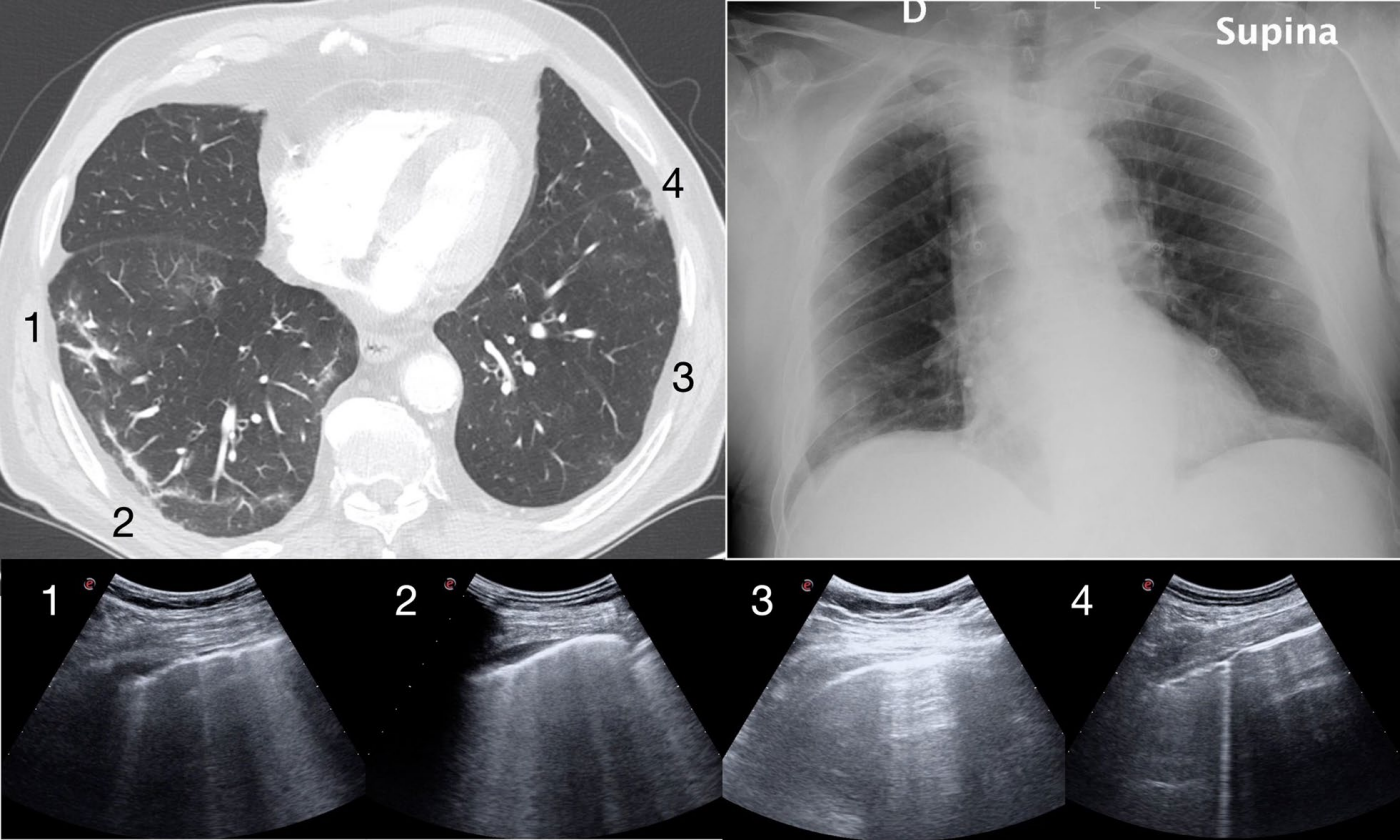
The case of an otherwise healthy 77-year-old man complaining of fever and cough for 7 days (*mild phenotype*). LUS and CXR were in disagreement. LUS showed a *high probability pattern* for COVID-19 pneumonia. The LUS scans reported in the bottom show the patchy and bilateral distribution of clusters of B-lines and light beam (1, 2, 4) with some irregularity of the pleural line (1, 4), alternating with normal A-lines pattern (3). CXR in the upper right panel was read *negative*. Corresponding CT scan in the upper left panel shows bilateral GGO and was considered *typical appearance*. The points numbered in the CT image correspond to the 4 LUS scans. Viral RT-PCR was positive. *LUS*   Lung Ultrasound, *CXR*  Chest Radiography, *COVID-19*  Corona Virus Disease 2019, *GGO*   Ground Glass Opacity, *CT*   Computed Tomography, *RT-PCR*  Reverse Transcriptase-Polymerase Chain Reaction

**Fig. 4:**
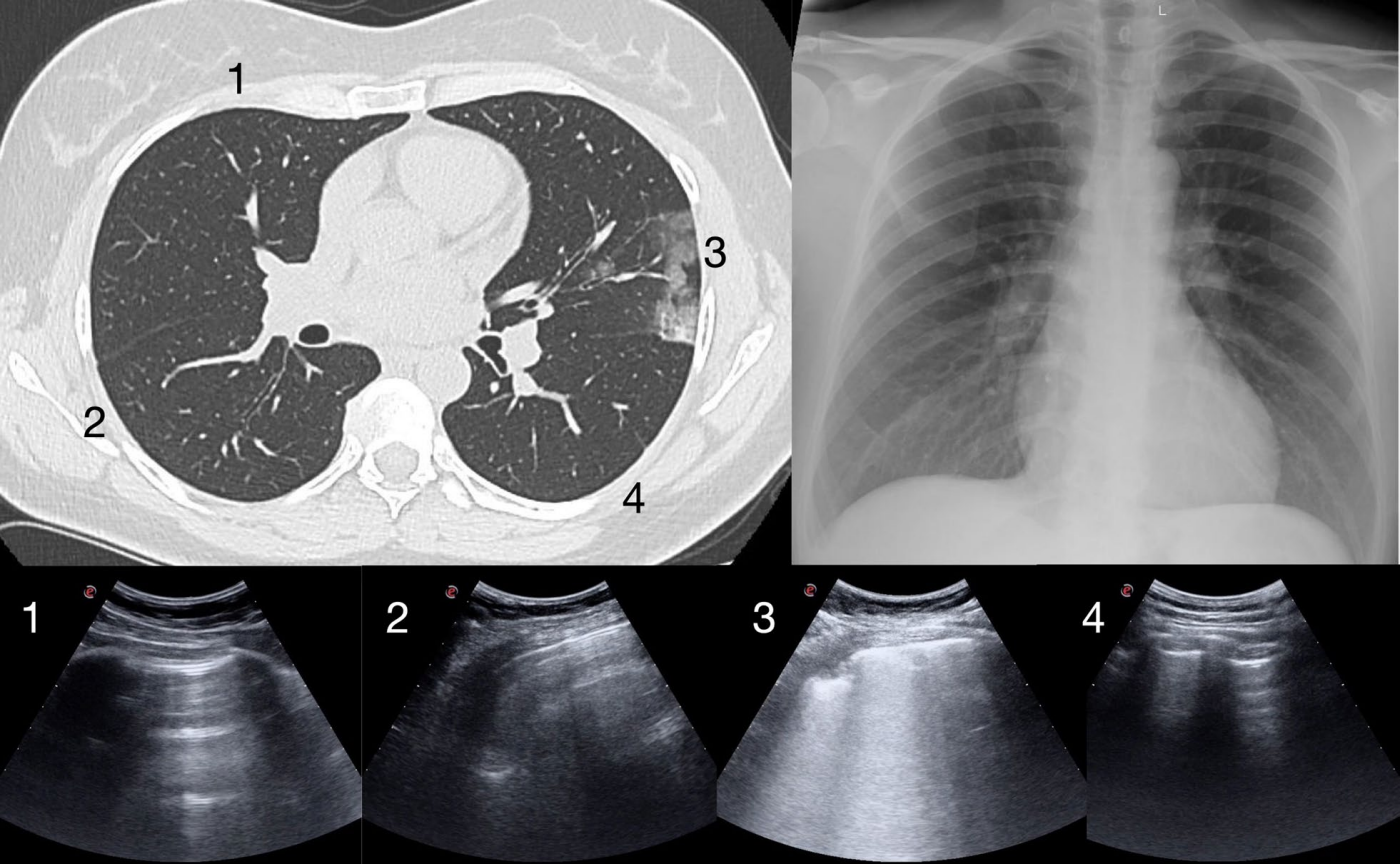
The case of an otherwise healthy 44-year-old woman complaining of malaise, anosmia, fever and cough for 4 days (*mild phenotype*). LUS and CXR were in disagreement. LUS showed an *intermediate probability pattern* for COVID-19 pneumonia. One of the LUS scans reported in the bottom shows an isolated area with B-lines, light beam and a peripheral consolidation (3). The other LUS scans show A-lines (1, 2, 4). CXR in the upper right panel was read *negative*. Corresponding CT scan in the upper left panel shows a limited peripheral area with GGO and a consolidation and was considered *indeterminate appearance*. The points numbered in the CT scan correspond to the 4 LUS scans. Viral RT-PCR was positive. *LUS*   Lung Ultrasound, *CXR*  Chest Radiography, *COVID-19*  Corona Virus Disease 2019, *GGO*   Ground Glass Opacity, *CT*   Computed Tomography, *RT-PCR*  Reverse Transcriptase-Polymerase Chain Reaction

*HighLUS and IntLUS with negative CXR*: in 18 cases HighLUS (*n* = 8) and IntLUS (*n = *10) corresponded to *negative* CXR readings (Figs. 3 and 4). Out of the 8 disagreement HighLUS cases, in one case, a CT study was performed that confirmed the *typical* appearance. All the other 7 cases were confirmed by positivity of RT-PCR. Out of the 10 IntLUS cases, in 3 cases, a CT study confirmed an *indeterminate* appearance and in other 3 cases, the RT-PCR was positive. In the remaining 4 cases, RT-PCR resulted negative.

*LowLUS*: in 6 cases showing disagreement, LowLUS corresponded to *indeterminate* CXR (*n* = 3) and *atypical* CXR (*n* = 3). Two of these patients performed CT scan that confirmed a *negative* appearance. One other was confirmed negative by the RT-PCR. The other 3 cases, of which one with *atypical* CXR and two *indeterminate* CXR, resulted RT-PCR-positive.

**Fig. 5:**
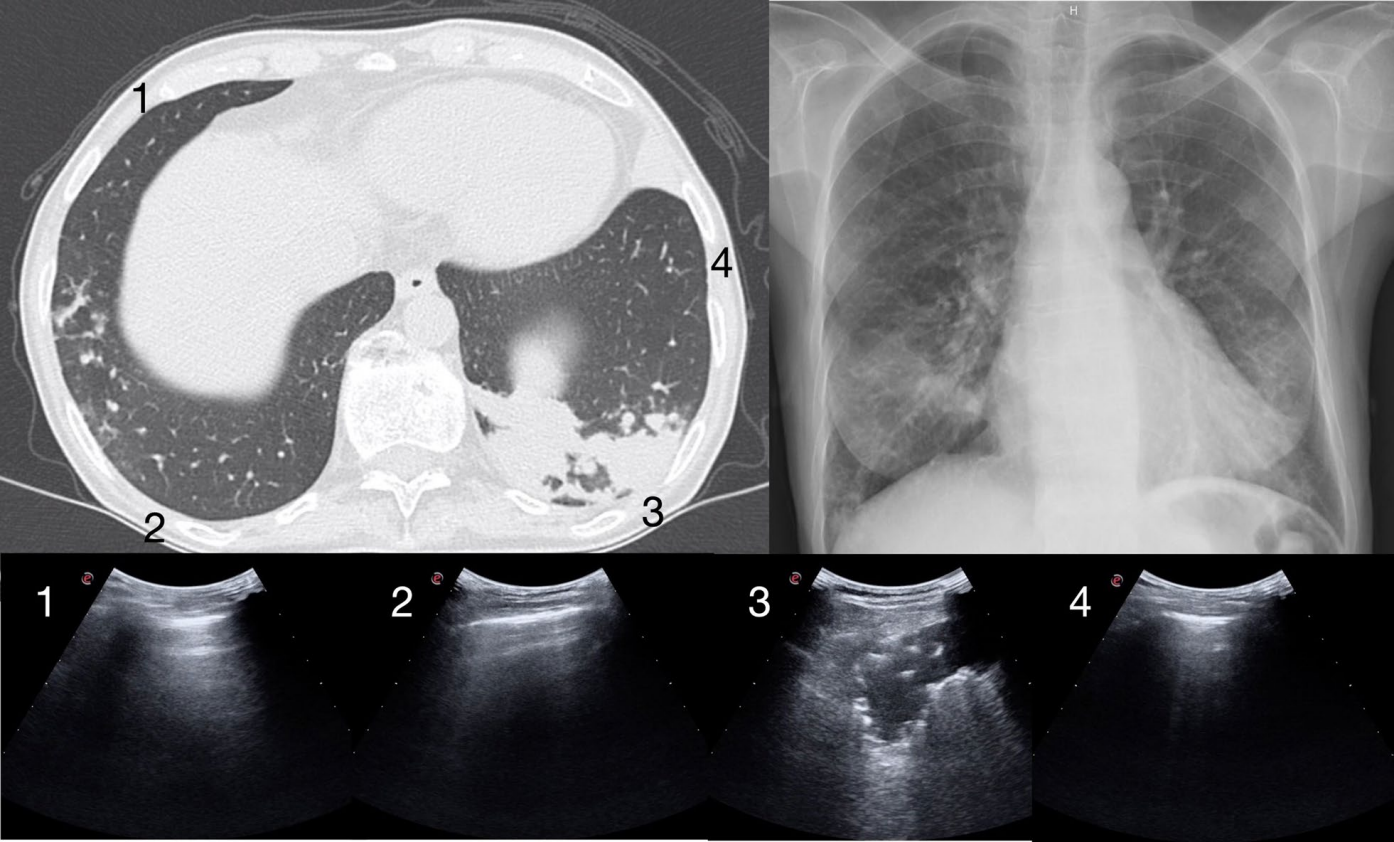
The case of a 48-year-old woman with a history of HCV related chronic hepatitis, complaining of fever and cough for two weeks (*mild phenotype*). LUS and CXR were in disagreement. LUS showed an *alternative pattern* to COVID-19 pneumonia. One of the LUS scans reported in the bottom shows a large lobar isolated consolidation with dynamic air bronchograms (3). The other LUS scans show A-lines (1, 2, 4). CXR in the upper right panel was read *typical* of COVID-19 pneumonia due to bilateral reticular alterations and GGO. Corresponding CT scan in the upper left panel shows a large consolidation with air bronchograms without significant GGO alterations and was confirmed *atypical appearance* (bacterial pneumonia) The points numbered in the CT scan correspond to the 4 LUS scans. Viral RT-PCR was negative. The patient recovered after antibiotic treatment. *LUS*   Lung Ultrasound, *CXR*  Chest Radiography, *COVID-19*  Corona Virus Disease 2019, *GGO*   Ground Glass Opacity, *CT*   Computed Tomography, *RT-PCR*  Reverse Transcriptase-Polymerase Chain Reaction

*AltLUS*: in 6 cases showing disagreement, AltLUS corresponded to *negative* CXR and in 4 of these cases, the CT study showed *atypical* appearance, confirming the LUS interpretation. In the remaining 2 cases, RT-PCR resulted positive in one and negative in the other. In 5 cases, AltLUS corresponded to *typical* CXR, but RT-PCR resulted negative in all and in two cases CT scan confirmed the *atypical* appearance (Fig. 5). In two cases, AltLUS corresponded to *indeterminate* CXR with a positive RT-PCR in one and a confirmation of *atypical* CT in the other.

## Discussion

Our study demonstrated a high rate of disagreement between LUS interpretation and CXR reading when the symptomatic patients are examined in the ED for suspected COVID-19 pneumonia during the pandemic surge. Disagreement between LUS and CXR was irrespective of the main patient’s characteristics, including sex, days of symptoms onset and clinical phenotype. The group with disagreement resulted only significantly slightly older.

The high rate of disagreement may be due to the classification in 4 different LUS patterns of probability. However, the LUS probability approach demonstrated highly practical when suspected cases are examined in the ED and helps to allocate the patients during the pandemic surge [1, 14]. Indeed, quite a similar classification is recommended for CT readings in COVID-19 pneumonia [19]. The LUS classification includes the possibility of interpreting an intermediate likelihood for COVID-19 pneumonia, corresponding to the “*indeterminate*” classification of the CT language. Like for CT, the LUS *intermediate* probability is assigned in cases of unilateral and mostly isolated interstitial signs. Indeed, to compare LUS probability patterns and CXR readings, it was necessary to introduce the possibility of an “*indeterminate*” reading also for the interpretation of the CXR exams, which may have increased LUS vs CXR disagreement. However, even mixing together *typical* and *indeterminate* CXR readings and *high* and *intermediate* probability LUS interpretation, the rate of disagreement still remained high.

Age may have a role, as an older age may imply an increase in confounding sonographic and radiologic signs. Indeed, the age of the group of disagreement cases was slightly but significantly older than in the other group. However, in other studies, CXR showed a low sensitivity in the early diagnosis of COVID-19 pneumonia irrespective of patient’s age, whereas sensitivity improved when symptoms had started from a longer time [16].

Based on our data, it is reasonable to hypothesize that LUS is a more powerful tool in the early diagnosis of COVID-19 pneumonia and that the high rate of disagreement is justified by a difference in diagnostic accuracy between LUS and CXR. This hypothesis is supported also by a more detailed analysis of our disagreement cases, based on the comparison with CT studies and results of the viral swab test. Our study was not specifically designed to investigate a systematic comparison between CT scan and LUS, but the subgroup of patients submitted to all the three imaging tools showed the striking superiority of LUS to CXR, based on a higher rate of agreement between CT vs LUS interpretation than CT vs CXR reading. When a CT scan was not performed, the results of RT-PCR demonstrated a high rate of confirmation of the LUS classification. Of course, this latter result should be considered cautiously as the viral test is useful to confirm the SARS-CoV-2 infection but not necessarily this latter condition is complicated by pneumonia. Thus, a negative chest imaging test may easily be coupled with a positive RT-PCR in case of SARS-CoV-2 infection not complicated by pneumonia. On the other side, it is also well known that the molecular RT-PCR is limited by quite a low sensitivity, especially when performed early in the first approach to suspected cases [20, 21]. It is common experience that hospitalized patients with a typical clinical picture and suggestive computed tomography (CT) pattern but repeatedly negative RT-PCR, have been considered and treated as COVID-19 pneumonia, particularly in the peak of the COVID-19 surge [2].

Other studies compared LUS and CXR in COVID-19, demonstrating a higher sensitivity of LUS [22, 23]. These studies used a binary classification of the interpretation of LUS based on the presence of generic artifactual abnormalities that, in our opinion, does not adapt to the real practice. Indeed, cases of COVID-19 pneumonia are characterized by non-specific ultrasound signs that can only allow assignment of degrees of probability after the analysis of their distribution. Thus, we strongly suggest comparing abilities of LUS and CXR in the definition of degrees of probability, more than assigning binary diagnoses of positive or negative COVID-19 [1, 7]. Moreover, there is a misconception on the utility of chest imaging that cannot be evaluated for the ability in allowing a definitive diagnosis of COVID-19 infection [24]. The role of chest imaging tools is limited to the diagnosis of pneumonia, which not necessarily complicates COVID-19 and, when confirmed, not always is caused by SARS-CoV-2. Only a combination of chest imaging, clinical data, RT-PCR and follow-up should guide patient’s management and safe assignment of differential diagnoses.

## Limitations

In our study, the CXR reading was performed retrospectively by expert radiologists. We did not consider the original reading performed in the ED by the radiologist in charge, which would had been more realistic of what happens in the early approach to patients during the surge. However, for intuitive reasons, the CXR readings on the field could not adhere to a strict standardization and the language used could not facilitate a systematic comparison with CT and LUS interpretations.

Another limitation of our study, was the lack of a systematic comparison with CT. Indeed, CT represents the gold standard in the diagnosis of COVID-19 pneumonia. Notwithstanding, the systematic application of CT studies is not ethical in the time of a COVID-19 pandemic surge, due mainly to the problem of overexposure of personnel outside the dedicated hospital areas and lack of clinical utility during the early management of the vast majority of COVID-19 cases [5]. We used the result of RT-PCR to select patients with and without infection. However, the diagnosis of pneumonia is independent from RT-PCR as COVID-19 not necessarily involves the lung and patients with pneumonia may present with negative RT-PCR. Thus, the results of our study should be read as purely descriptive in a moment of extremely high COVID-19 prevalence, and cannot be used for a definitive statistical diagnostic analysis.

A further limitation may be considered the application of bedside CXR antero-posterior projections. It is well known the limitation of this view in the evaluation of the pulmonary parenchyma. However, due to the necessity of limiting the possibility of hospital cross-infections, the bedside CXR is the standard of care in the time of the COVID-19 pandemic surge.

Finally, the study was conducted in a single center where LUS is used as a standard bedside diagnostic tool from many years. In other centers, the quality of LUS performance may vary depending on the level of experience of the operators.

## Conclusion

Our descriptive study showed a high rate of disagreement between LUS and CXR performed during the early evaluation of patients suspected of COVID-19. The results of this study support the idea that LUS is superior to CXR in the first approach to COVID-19.

## Data Availability

Collected data are available in the form of an Excel worksheet on request to Giovanni Volpicelli, giovi.volpicelli@gmail.com. Data are listed anonymously. The data set was analyzed by all the co-authors.

## Abbreviations

COVID-19: Corona Virus Disease 2019
SARS-CoV-2: Severe Acute Respiratory Syndrome Corona Virus-2
CT: Computed Tomography
GGO: Ground Glass Opacity
LUS: Lung Ultrasound
ED: Emergency Department
CXR: Chest Radiography
RT-PCR: Reverse Transcriptase-Polymerase Chain Reaction
PaO_2_: Partial Pressure of Oxygen in arterial blood
Sat.O_2_: Oxygen Saturation
FiO_2_: Fraction of inspired Oxygen
HighLUS: High Probability Lung Ultrasound pattern
IntLUS: Intermediate Probability Lung Ultrasound pattern
AltLUS: Alternative Probability Lung Ultrasound pattern
LowLUS: Low Probability Lung Ultrasound pattern
kV: kilovoltage
mAs: milliampere-second
SD: Standard Deviation

## References

[1] Volpicelli G, Lamorte A, Villén T (2020). What's new in lung ultrasound during the COVID-19 pandemic. Intensive Care Med.

[2] Ai T, Yang Z, Hou H, Zhan C, Chen C, Lv W, Tao Q, Sun Z, Xia L (2020). Correlation of chest CT and RT-PCR testing for coronavirus disease 2019 (COVID-19) in China: A Report of 1014 Cases. Radiology.

[3] Han R, Huang L, Jiang H, Dong J, Peng H, Zhang D (2020). Early clinical and CT manifestations of coronavirus disease 2019 (COVID-19) Pneumonia. AJR Am J Roentgenol.

[4] Hope MD, Raptis CA, Shah A, Hammer MM, Henry TS, six signatories (2020). A role for CT in COVID-19? What data really tell us so far. Lancet.

[5] Revel MP, Parkar AP, Prosch H, Silva M, Sverzellati N, Gleeson F, Brady A, European Society of Radiology (ESR) and the European Society of Thoracic Imaging (ESTI) (2020). COVID-19 patients and the radiology department - advice from the European Society of Radiology (ESR) and the European Society of Thoracic Imaging (ESTI). Eur Radiol.

[6] Volpicelli G, Gargani L (2020). Sonographic signs and patterns of COVID-19 pneumonia. Ultrasound J.

[7] Millington SJ, Koenig S, Mayo P, Volpicelli G (2021). Lung ultrasound for patients with coronavirus disease 2019 pulmonary disease. Chest.

[8] Zieleskiewicz L, Markarian T, Lopez A, Taguet C, Mohammedi N, Boucekine M, Baumstarck K, Besch G, Mathon G, Duclos G, Bouvet L, Michelet P, Allaouchiche B, Chaumoître K, Di Bisceglie M, Leone M, AZUREA Network (2020). Comparative study of lung ultrasound and chest computed tomography scan in the assessment of severity of confirmed COVID-19 pneumonia. Intensive Care Med.

[9] Pivetta E, Goffi A, Tizzani M, Locatelli SM, Porrino G, Losano I, Leone D, Calzolari G, Vesan M, Steri F, Ardito A, Capuano M, Maria G, Giulia S, Dutto S, Avolio M, Cavallo R, Bartalucci A, Paglieri C, Morello F, Richiardi L, Maule M, Lupia E (2020). Lung ultrasound for the diagnosis of SARS-CoV-2 pneumonia in the emergency department. Ann Emerg Med.

[10] Ippolito D, Pecorelli A, Maino C, Capodaglio C, Mariani I, Giandola T, Gandola D, Bianco I, Ragusi M, Talei Franzesi C, Corso R, Sironi S (2020). Diagnostic impact of bedside chest X-ray features of 2019 novel coronavirus in the routine admission at the emergency department: case series from Lombardy region. Eur J Radiol.

[11] Pakray A, Walker D, Figacz A, Kilanowski S, Rhodes C, Doshi S, Coffey M (2020). Imaging evaluation of COVID-19 in the emergency department. Emergency Radiol.

[12] Schiaffino S, Tritella S, Cozzi A, Carriero S, Blandi L, Ferraris L, Sardanelli F (2020). Diagnostic Performance of Chest X-Ray for COVID-19 Pneumonia During the SARS-CoV-2 Pandemic in Lombardy. Italy J Thorac Imaging.

[13] Vespro V, Andrisani MC, Fusco S, Di Meglio L, Plensich G, Scarabelli A, Stellato E, Ierardi AM, Scudeller L, Coppola A, Gori A, Pesenti A, Grasselli G, Aliberti S, Blasi F, Villa C, Ippolito S, Pirrò B, Damiani G, Galli M, Rizzardini G, Catena E, Orlandi MA, Magnani S, Cipolla G, Ianniello AA, Petrillo M, Xhepa G, Scamporrino A, Cazzulani A, Carrafiello G (2020). Chest X-ray findings in a large cohort of 1117 patients with SARS-CoV-2 infection: a multicenter study during COVID-19 outbreak in Italy. Intern Emerg Med.

[14] Volpicelli G, Gargani L, Perlini S, Spinelli S, Barbieri G, Lanotte A, Casasola GG, Nogué Bou R, Lamorte A, Agricola E, Villén T, Deol PS, Nazerian P, Corradi F, Stefanone V, Fraga DN, Navalesi P, Ferre R, Boero E, Martinelli G, Cristoni L, Perani C, Vetrugno L, McDermott C, Miralles-Aguiar F, Secco G, Zattera C, Salinaro F, Grignaschi A, Boccatonda A, Giostra F, Nogué Infante M, Covella M, Ingallina G, Burkert J, Frumento P, Forfori F, Ghiadoni L, on behalf of the International Multicenter Study on LUS in COVID-19 (2021) Lung ultrasound for the early diagnosis of COVID-19 pneumonia: an international multicenter study. Intensive Care Med **(in press)** 10.1007/s00134-021-06373-7 PMC7980130 33743018

[15] Gargani L, Soliman-Aboumarie H, Volpicelli G, Corradi F, Pastore MC, Cameli M (2020). Why, when, and how to use lung ultrasound during the COVID-19 pandemic: enthusiasm and caution. Eur Heart J Cardiovasc Imaging.

[16] Vancheri SG, Savietto G, Ballati F, Maggi A, Canino C, Bortolotto C, Valentini A, Dore R, Stella GM, Corsico AG, Iotti GA, Mojoli F, Perlini S, Bruno R, Preda L (2020). Radiographic findings in 240 patients with COVID-19 pneumonia: time-dependence after the onset of symptoms. Eur Radiol.

[17] Volpicelli G, Elbarbary M, Blaivas M, Lichtenstein DA, Mathis G, Kirkpatrick AW, Melniker L, Gargani L, Noble VE, Via G, Dean A, Tsung JW, Soldati G, Copetti R, Bouhemad B, Reissig A, Agricola E, Rouby JJ, Arbelot C, Liteplo A, Sargsyan A, Silva F, Hoppmann R, Breitkreutz R, Seibel A, Neri L, Storti E, Petrovic T, International Liaison Committee on Lung Ultrasound (ILC-LUS) for International Consensus Conference on Lung Ultrasound (ICC-LUS) (2012). International evidence-based recommendations for point-of-care lung ultrasound. Intensive Care Med.

[18] Hansell DM, Bankier AA, MacMahon H, McLoud TC, Muller NL, Remy J (2008). Fleischner Society: glossary of terms for thoracic imaging. Radiology.

[19] Simpson S, Kay FU, Abbara S, Bhalla S, Chung JH, Chung M, Henry TS, Kanne JP, Kligerman S, Ko JP, Litt H (2020). Radiological Society of North America Expert Consensus Statement on Reporting Chest CT Findings Related to COVID-19. Endorsed by the Society of Thoracic Radiology, the American College of Radiology, and RSNA. J Thorac Imaging.

[20] Li Y, Yao L, Li J, Chen L, Song Y, Cai Z, Yang C (2020). Stability issues of RT-PCR testing of SARS-CoV-2 for hospitalized patients clinically diagnosed with COVID-19. J Med Virol.

[21] Arevalo-Rodriguez I, Buitrago-Garcia D, Simancas-Racines D, Zambrano-Achig P, Del Campo R, Ciapponi A, Sued O, Martínez-García L, Rutjes A, Low N, Bossuyt PM, Perez-Molina JA, Zamora J (2020) False-negative results of initial RT-PCR assays for COVID-19: a systematic review. https://www.medrxiv.org/content/10.1101/2020.04.16.20066787v1 10.1371/journal.pone.0242958 PMC7728293 33301459

[22] Shumilov E, Hosseini ASA, Petzold G, Treiber H, Lotz J, Ellenrieder V, Kunsch S, Neesse A (2020). Comparison of Chest Ultrasound and Standard X-Ray Imaging in COVID-19 Patients. Ultrasound Int Open.

[23] Pare JR, Camelo I, Mayo KC, Leo MM, Dugas JN, Nelson KP, Baker WE, Shareef F, Mitchell PM, Schechter-Perkins EM (2020). Point-of-care lung ultrasound is more sensitive than chest radiograph for evaluation of COVID-19. West J Emerg Med.

[24] Islam N, Salameh JP, Leeflang MM, Hooft L, McGrath TA, van der Pol CB, Frank RA, Kazi S, Prager R, Hare SS, Dennie C, Spijker R, Deeks JJ, Dinnes J, Jenniskens K, Korevaar DA, Cohen JF, Van den Bruel A, Takwoingi Y, van de Wijgert J, Wang J, McInnes MD, Cochrane COVID-19 Diagnostic Test Accuracy Group (2020). Thoracic imaging tests for the diagnosis of COVID-19. Cochrane Database Syst Rev.

